# Supplementary material for: Modulation of alpha oscillations by attention is predicted by hemispheric asymmetry of subcortical regions
Source: eLife. 2024 Jul 17;12:RP91650. doi: 10.7554/eLife.91650 (PMC11254381; doi:10.7554/eLife.91650)
Supplement: Supplementary file 1. — The selected model, with lowest AIC, is marked in green. [file elife-91650-supp1.docx]

Supplementary file 1. Akaike Information Criterion (AIC) and Bayesian Information Criterion (BIC) values for all possible combinations of regressors (Lateralized Volume of subcortical structures). The selected model, with lowest AIC, is marked in green.

| *Regression* | Comb. 1 | Comb. 2 | Comb. 3 | Comb. 4 | Comb. 5 | Comb. 6 | Comb. 7 | Comb. 8 | Comb. 9 | Comb. 10 | Comb. 11 | Comb. 12 | Comb. 13 | Comb. 14 | Comb. 15 | Comb. 16 | Comb. 17 | Comb. 18 | Comb. 19 | Comb. 20 | Comb. 21 | Model criterion |
| --- | --- | --- | --- | --- | --- | --- | --- | --- | --- | --- | --- | --- | --- | --- | --- | --- | --- | --- | --- | --- | --- | --- |
| $\boldsymbol{HLM \sim}\boldsymbol{\beta}_{\boldsymbol{0}}\boldsymbol{+}\boldsymbol{\beta}_{\boldsymbol{LV}_{\mathbf{1}}}$ | -81.63 | -77.78 | -73.75 | -78.62 | -71.94 | -73.18 | -71.48 | NaN | NaN | NaN | NaN | NaN | NaN | NaN | NaN | NaN | NaN | NaN | NaN | NaN | NaN | **AIC** |
|  | -77.33 | -73.48 | -69.45 | -74.32 | -67.64 | -68.88 | -67.18 | NaN | NaN | NaN | NaN | NaN | NaN | NaN | NaN | NaN | NaN | NaN | NaN | NaN | NaN | **BIC** |
| $\boldsymbol{HLM \sim}\boldsymbol{\beta}_{\boldsymbol{0}}\boldsymbol{+}\boldsymbol{\beta}_{\boldsymbol{LV}_{\mathbf{1}}} \boldsymbol{+}\boldsymbol{\beta}_{\boldsymbol{LV}_{\mathbf{2}}}$ | -86.72 | -79.93 | -82.66 | -77.88 | -78.04 | -78.00 | -76.42 | -81.27 | -75.82 | -75.83 | -73.89 | -77.41 | -70.41 | -71.36 | -69.94 | -74.79 | -76.05 | -74.63 | -70.08 | -68.23 | -69.30 | **AIC** |
|  | -81.11 | -74.32 | -77.05 | -72.28 | -72.44 | -72.39 | -70.82 | -75.66 | -70.22 | -70.23 | -68.29 | -71.81 | -64.81 | -65.75 | -64.33 | -69.19 | -70.44 | -69.03 | -64.48 | -62.63 | -63.70 | **BIC** |
| $\boldsymbol{HLM \sim}\boldsymbol{\beta}_{\boldsymbol{0}}\boldsymbol{+}\boldsymbol{\beta}_{\boldsymbol{LV}_{\mathbf{1}}}\boldsymbol{+}\boldsymbol{\beta}_{\boldsymbol{LV}_{\mathbf{2}}}\boldsymbol{+}\boldsymbol{\beta}_{\boldsymbol{LV}_{\mathbf{3}}}$ | -85.30 | -87.54 | -82.79 | -82.96 | -82.64 | -81.00 | -76.18 | -76.19 | -76.29 | -78.86 | -79.00 | -78.73 | -74.25 | -74.24 | -74.32 | -79.67 | -74.61 | -74.06 | -72.55 | -78.51 | -78.88 | **AIC** |
|  | -78.46 | -80.71 | -75.95 | -75.13 | -75.81 | -74.17 | -69.34 | -69.35 | -69.46 | -72.02 | -72.17 | -71.90 | -67.42 | -57.40 | -67.49 | -72.84 | -67.77 | -67.22 | -65.72 | -71.68 | -72.05 | **BIC** |
| $\boldsymbol{HLM \sim}\boldsymbol{\beta}_{\boldsymbol{0}}\boldsymbol{+}\boldsymbol{\beta}_{\boldsymbol{LV}_{\mathbf{1}}}\boldsymbol{+}\boldsymbol{\beta}_{\boldsymbol{LV}_{\mathbf{2}}}\boldsymbol{+}\boldsymbol{\beta}_{\boldsymbol{LV}_{\mathbf{3}}}\boldsymbol{+}\boldsymbol{\beta}_{\boldsymbol{LV}_{\mathbf{4}}}$ | -85.52 | -81.42 | -81.27 | -81.21 | -83.50 | -83.73 | -83.30 | -79.33 | -78.80 | -78.85 | -77.26 | -77.72 | -77.09 | -72.43 | -72.54 | -72.49 | -75.15 | -74.95 | -75.04 | -70.57 | -76.78 | **AIC** |
|  | -77.52 | -73.43 | -73.28 | -73.21 | -75.50 | -75.74 | -75.31 | -71.33 | -70.80 | -70.86 | -69.27 | -69.73 | -69.10 | -64.44 | -64.55 | -64.45 | -67.15 | -66.96 | -67.04 | -62.58 | -68.78 | **BIC** |
| $\boldsymbol{HLM \sim}\boldsymbol{\beta}_{\boldsymbol{0}}\boldsymbol{+}\boldsymbol{\beta}_{\boldsymbol{LV}_{\mathbf{1}}}\boldsymbol{+}\boldsymbol{\beta}_{\boldsymbol{LV}_{\mathbf{2}}}\boldsymbol{+}\boldsymbol{\beta}_{\boldsymbol{LV}_{\mathbf{3}}}\boldsymbol{+}\boldsymbol{\beta}_{\boldsymbol{LV}_{\mathbf{4}}}\boldsymbol{+}\boldsymbol{\beta}_{\boldsymbol{LV}_{\mathbf{5}}}$ | -81.51 | -81.90 | -81.30 | -77.65 | -77.41 | -77.17 | -79.95 | -79.32 | -79.49 | -75.29 | -73.88 | -73.36 | -73.75 | -68.77 | -71.22 | -75.31 | -72.78 | -73.87 | -68.85 | -72.63 | -67.74 | **AIC** |
|  | -72.44 | -72.83 | -72.24 | -68.58 | -68.34 | -68.01 | -70.88 | -70.25 | -70.42 | -66.22 | -64.81 | -64.29 | -64.68 | -59.70 | -62.15 | -66.24 | -63.71 | -64.79 | -59.78 | -63.56 | -58.67 | **BIC** |
| $\boldsymbol{HLM \sim}\boldsymbol{\beta}_{\boldsymbol{0}}\boldsymbol{+}\boldsymbol{\beta}_{\boldsymbol{LV}_{\mathbf{1}}}\boldsymbol{+}\boldsymbol{\beta}_{\boldsymbol{LV}_{\mathbf{2}}}\boldsymbol{+}\boldsymbol{\beta}_{\boldsymbol{LV}_{\mathbf{3}}}\boldsymbol{+}\boldsymbol{\beta}_{\boldsymbol{LV}_{\mathbf{4}}}\boldsymbol{+}\boldsymbol{\beta}_{\boldsymbol{LV}_{\mathbf{5}}}\boldsymbol{+}\boldsymbol{\beta}_{\boldsymbol{LV}_{\mathbf{6}}}$ | -78.13 | -77.37 | -77.70 | -73.63 | -75.76 | -69.96 | -71.25 | NaN | NaN | NaN | NaN | NaN | NaN | NaN | NaN | NaN | NaN | NaN | NaN | NaN | NaN | **AIC** |
|  | -68.07 | -67.30 | -67.63 | -63.56 | -65.70 | -59.89 | -61.19 | NaN | NaN | NaN | NaN | NaN | NaN | NaN | NaN | NaN | NaN | NaN | NaN | NaN | NaN | **BIC** |
| $\boldsymbol{HLM \sim}\boldsymbol{\beta}_{\boldsymbol{0}}\boldsymbol{+}\boldsymbol{\beta}_{\boldsymbol{LV}_{\mathbf{1}}}\boldsymbol{+}\boldsymbol{\beta}_{\boldsymbol{LV}_{\mathbf{2}}}\boldsymbol{+} \boldsymbol{\beta}_{\boldsymbol{LV}_{\mathbf{3}}}\boldsymbol{+}\boldsymbol{\beta}_{\boldsymbol{LV}_{\mathbf{4}}}\boldsymbol{+}\boldsymbol{\beta}_{\boldsymbol{LV}_{\mathbf{5}}}\boldsymbol{+}\boldsymbol{\beta}_{\boldsymbol{LV}_{\mathbf{6}}}\boldsymbol{+}\boldsymbol{\beta}_{\boldsymbol{LV}_{\mathbf{7}}}$ | -74.00 | NaN | NaN | NaN | NaN | NaN | NaN | NaN | NaN | NaN | NaN | NaN | NaN | NaN | NaN | NaN | NaN | NaN | NaN | NaN | NaN | **AIC** |
|  | -63.01 | NaN | NaN | NaN | NaN | NaN | NaN | NaN | NaN | NaN | NaN | NaN | NaN | NaN | NaN | NaN | NaN | NaN | NaN | NaN | NaN | **BIC** |

| *Regression* | Comb. 22 | Comb. 23 | Comb. 24 | Comb. 25 | Comb. 26 | Comb. 27 | Comb. 28 | Comb. 29 | Comb. 30 | Comb. 31 | Comb. 32 | Comb. 33 | Comb. 34 | Comb. 35 | Model criterion |
| --- | --- | --- | --- | --- | --- | --- | --- | --- | --- | --- | --- | --- | --- | --- | --- |
| $\boldsymbol{HLM \sim}\boldsymbol{\beta}_{\boldsymbol{0}}\boldsymbol{+}\boldsymbol{\beta}_{\boldsymbol{LV}_{\mathbf{1}}}$ | NaN | NaN | NaN | NaN | NaN | NaN | NaN | NaN | NaN | NaN | NaN | NaN | NaN | NaN | **AIC** |
|  | NaN | NaN | NaN | NaN | NaN | NaN | NaN | NaN | NaN | NaN | NaN | NaN | NaN | NaN | **BIC** |
| $\boldsymbol{HLM \sim}\boldsymbol{\beta}_{\boldsymbol{0}}\boldsymbol{+}\boldsymbol{\beta}_{\boldsymbol{LV}_{\mathbf{1}}} \boldsymbol{+}\boldsymbol{\beta}_{\boldsymbol{LV}_{\mathbf{2}}}$ | NaN | NaN | NaN | NaN | NaN | NaN | NaN | NaN | NaN | NaN | NaN | NaN | NaN | NaN | **AIC** |
|  | NaN | NaN | NaN | NaN | NaN | NaN | NaN | NaN | NaN | NaN | NaN | NaN | NaN | NaN | **BIC** |
| $\boldsymbol{HLM \sim}\boldsymbol{\beta}_{\boldsymbol{0}}\boldsymbol{+}\boldsymbol{\beta}_{\boldsymbol{LV}_{\mathbf{1}}}\boldsymbol{+}\boldsymbol{\beta}_{\boldsymbol{LV}_{\mathbf{2}}}\boldsymbol{+}\boldsymbol{\beta}_{\boldsymbol{LV}_{\mathbf{3}}}$ | -77.34 | -74.59 | -71.90 | -71.93 | -73.54 | -75.43 | -73.45 | -68.27 | -66.72 | -67.51 | -72.40 | -70.89 | -72.05 | -66.31 | **AIC** |
|  | -70.50 | -67.75 | -65.07 | -65.09 | -66.71 | -68.59 | -66.61 | -61.43 | -59.89 | -60.67 | -65.57 | -64.05 | -65.21 | -59.48 | **BIC** |
| $\boldsymbol{HLM \sim}\boldsymbol{\beta}_{\boldsymbol{0}}\boldsymbol{+}\boldsymbol{\beta}_{\boldsymbol{LV}_{\mathbf{1}}}\boldsymbol{+}\boldsymbol{\beta}_{\boldsymbol{LV}_{\mathbf{2}}}\boldsymbol{+}\boldsymbol{\beta}_{\boldsymbol{LV}_{\mathbf{3}}}\boldsymbol{+}\boldsymbol{\beta}_{\boldsymbol{LV}_{\mathbf{4}}}$ | -77.76 | -75.76 | -72.83 | -70.69 | -70.19 | -76.72 | -74.48 | -74.97 | -70.58 | -71.67 | -69.66 | -71.44 | -64.53 | -68.46 | **AIC** |
|  | -69.76 | -67.77 | -64.84 | -62.70 | -62.19 | -68.73 | -66.49 | -66.98 | -62.59 | -63.68 | -61.67 | -63.44 | -56.53 | -60.47 | **BIC** |
| $\boldsymbol{HLM \sim}\boldsymbol{\beta}_{\boldsymbol{0}}\boldsymbol{+}\boldsymbol{\beta}_{\boldsymbol{LV}_{\mathbf{1}}}\boldsymbol{+}\boldsymbol{\beta}_{\boldsymbol{LV}_{\mathbf{2}}}\boldsymbol{+}\boldsymbol{\beta}_{\boldsymbol{LV}_{\mathbf{3}}}\boldsymbol{+}\boldsymbol{\beta}_{\boldsymbol{LV}_{\mathbf{4}}}\boldsymbol{+}\boldsymbol{\beta}_{\boldsymbol{LV}_{\mathbf{5}}}$ | NaN | NaN | NaN | NaN | NaN | NaN | NaN | NaN | NaN | NaN | NaN | NaN | NaN | NaN | **AIC** |
|  | NaN | NaN | NaN | NaN | NaN | NaN | NaN | NaN | NaN | NaN | NaN | NaN | NaN | NaN | **BIC** |
| $\boldsymbol{HLM \sim}\boldsymbol{\beta}_{\boldsymbol{0}}\boldsymbol{+}\boldsymbol{\beta}_{\boldsymbol{LV}_{\mathbf{1}}}\boldsymbol{+}\boldsymbol{\beta}_{\boldsymbol{LV}_{\mathbf{2}}}\boldsymbol{+}\boldsymbol{\beta}_{\boldsymbol{LV}_{\mathbf{3}}}\boldsymbol{+}\boldsymbol{\beta}_{\boldsymbol{LV}_{\mathbf{4}}}\boldsymbol{+}\boldsymbol{\beta}_{\boldsymbol{LV}_{\mathbf{5}}}\boldsymbol{+}\boldsymbol{\beta}_{\boldsymbol{LV}_{\mathbf{6}}}$ | NaN | NaN | NaN | NaN | NaN | NaN | NaN | NaN | NaN | NaN | NaN | NaN | NaN | NaN | **AIC** |
|  | NaN | NaN | NaN | NaN | NaN | NaN | NaN | NaN | NaN | NaN | NaN | NaN | NaN | NaN | **BIC** |
| $\boldsymbol{HLM \sim}\boldsymbol{\beta}_{\boldsymbol{0}}\boldsymbol{+}\boldsymbol{\beta}_{\boldsymbol{LV}_{\mathbf{1}}}\boldsymbol{+}\boldsymbol{\beta}_{\boldsymbol{LV}_{\mathbf{2}}}\boldsymbol{+}\boldsymbol{\beta}_{\boldsymbol{LV}_{\mathbf{3}}}\boldsymbol{+}\boldsymbol{\beta}_{\boldsymbol{LV}_{\mathbf{4}}}\boldsymbol{+}\boldsymbol{\beta}_{\boldsymbol{LV}_{\mathbf{5}}}\boldsymbol{+}\boldsymbol{\beta}_{\boldsymbol{LV}_{\mathbf{6}}}\boldsymbol{+}\boldsymbol{\beta}_{\boldsymbol{LV}_{\mathbf{7}}}$ | NaN | NaN | NaN | NaN | NaN | NaN | NaN | NaN | NaN | NaN | NaN | NaN | NaN | NaN | **AIC** |
|  | NaN | NaN | NaN | NaN | NaN | NaN | NaN | NaN | NaN | NaN | NaN | NaN | NaN | NaN | **BIC** |

| $\boldsymbol{HLM \sim}\boldsymbol{\beta}_{\boldsymbol{0}}\boldsymbol{+}\boldsymbol{\beta}_{\boldsymbol{LV}_{\mathbf{1}}}\boldsymbol{+}\boldsymbol{\beta}_{\boldsymbol{LV}_{\mathbf{2}}}\boldsymbol{+}\boldsymbol{\beta}_{\boldsymbol{LV}_{\mathbf{3}}}\boldsymbol{+}\boldsymbol{\beta}_{\boldsymbol{LV}_{\mathbf{4}}}\boldsymbol{+}\boldsymbol{\beta}_{\boldsymbol{LV}_{\mathbf{5}}}$ | NaN | NaN | NaN | NaN | NaN | NaN | NaN | NaN | NaN | NaN | NaN | NaN | NaN | NaN |
| --- | --- | --- | --- | --- | --- | --- | --- | --- | --- | --- | --- | --- | --- | --- |
| $\boldsymbol{HLM \sim}\boldsymbol{\beta}_{\boldsymbol{0}}\boldsymbol{+}\boldsymbol{\beta}_{\boldsymbol{LV}_{\mathbf{1}}}\boldsymbol{+}\boldsymbol{\beta}_{\boldsymbol{LV}_{\mathbf{2}}}\boldsymbol{+}\boldsymbol{\beta}_{\boldsymbol{LV}_{\mathbf{3}}}\boldsymbol{+}\boldsymbol{\beta}_{\boldsymbol{LV}_{\mathbf{4}}}\boldsymbol{+}\boldsymbol{\beta}_{\boldsymbol{LV}_{\mathbf{5}}}\boldsymbol{+}\boldsymbol{\beta}_{\boldsymbol{LV}_{\mathbf{6}}}$ | NaN | NaN | NaN | NaN | NaN | NaN | NaN | NaN | NaN | NaN | NaN | NaN | NaN | NaN |
| $\boldsymbol{HLM \sim}\boldsymbol{\beta}_{\boldsymbol{0}}\boldsymbol{+}\boldsymbol{\beta}_{\boldsymbol{LV}_{\mathbf{1}}}\boldsymbol{+}\boldsymbol{\beta}_{\boldsymbol{LV}_{\mathbf{2}}}\boldsymbol{+}\boldsymbol{\beta}_{\boldsymbol{LV}_{\mathbf{3}}}\boldsymbol{+}\boldsymbol{\beta}_{\boldsymbol{LV}_{\mathbf{4}}}\boldsymbol{+}\boldsymbol{\beta}_{\boldsymbol{LV}_{\mathbf{5}}}\boldsymbol{+}\boldsymbol{\beta}_{\boldsymbol{LV}_{\mathbf{6}}}\boldsymbol{+}\boldsymbol{\beta}_{\boldsymbol{LV}_{\mathbf{7}}}$ | NaN | NaN | NaN | NaN | NaN | NaN | NaN | NaN | NaN | NaN | NaN | NaN | NaN | NaN |
