# Supplementary material for: Modulation of alpha oscillations by attention is predicted by hemispheric asymmetry of subcortical regions
Source: eLife. 2024 Jul 17;12:RP91650. doi: 10.7554/eLife.91650 (PMC11254381; doi:10.7554/eLife.91650)
Supplement: Supplementary file 2. — The selected model is marked in green. [file elife-91650-supp2.docx]

Supplementary file 2. The combination of structures for each regression model above. The selected model is marked in green.

| *Regression* | Comb. 1 | Comb. 2 | Comb. 3 | Comb. 4 | Comb. 5 | Comb. 6 | Comb. 7 | Comb. 8 | Comb. 9 | Comb. 10 | Comb. 11 | Comb. 12 | Comb. 13 | Comb. 14 | Comb. 15 | Comb. 16 | Comb. 17 | Comb. 18 | Comb. 19 | Comb. 20 | Comb. 21 |
| --- | --- | --- | --- | --- | --- | --- | --- | --- | --- | --- | --- | --- | --- | --- | --- | --- | --- | --- | --- | --- | --- |
| $\boldsymbol{HLM \sim}\boldsymbol{\beta}_{\boldsymbol{0}}\boldsymbol{+}\boldsymbol{\beta}_{\boldsymbol{LV}_{\mathbf{1}}}$ | Th | CN | Put. | GP | Hipp. | Amyg. | Acc. |  |  |  |  |  |  |  |  |  |  |  |  |  |  |
| $\boldsymbol{HLM \sim}\boldsymbol{\beta}_{\boldsymbol{0}}\boldsymbol{+}\boldsymbol{\beta}_{\boldsymbol{LV}_{\mathbf{1}}} \boldsymbol{+}\boldsymbol{\beta}_{\boldsymbol{LV}_{\mathbf{2}}}$ | Th + CN | Th + Put. | Th + GP | Th + Hipp. | Th + Amyg. | Th + Acc. | CN + Put. | CN + GP | CN + Hipp. | CN + Amyg. | CN + Acc. | Put. + GP | Put. + Hipp. | Put. + Amyg. | Put. + Acc. | GP + Hipp. | GP + Amyg. | GP + Acc. | Hipp. + Amyg. | Hipp. + Acc. | Amyg. + Acc. |
| $\boldsymbol{HLM \sim}\boldsymbol{\beta}_{\boldsymbol{0}}\boldsymbol{+}\boldsymbol{\beta}_{\boldsymbol{LV}_{\mathbf{1}}}\boldsymbol{+}\boldsymbol{\beta}_{\boldsymbol{LV}_{\mathbf{2}}}\boldsymbol{+}\boldsymbol{\beta}_{\boldsymbol{LV}_{\mathbf{3}}}$ | Th + CN + Put. | Th + CN + GP | Th + CN + Hipp. | Th + CN + Amyg. | Th + CN + Acc. | Th + Put. + GP | Th + Put. + Hipp. | Th + Put. + Amyg. | Th + Put. + Acc. | Th + GP + Hipp. | Th + GP + Amyg. | Th + GP + Acc. | Th + Hipp. + Amyg. | Th + Hipp. + Acc. | Th + Amyg. + Acc. | CN + Put. + GP | CN + Put. + Hipp. | CN + Put. + Amyg. | CN + Put. + Acc. | CN + GP + Hipp. | CN + GP + Amyg. |
| $\boldsymbol{HLM \sim}\boldsymbol{\beta}_{\boldsymbol{0}}\boldsymbol{+}\boldsymbol{\beta}_{\boldsymbol{LV}_{\mathbf{1}}}\boldsymbol{+}\boldsymbol{\beta}_{\boldsymbol{LV}_{\mathbf{2}}}\boldsymbol{+}\boldsymbol{\beta}_{\boldsymbol{LV}_{\mathbf{3}}}\boldsymbol{+}\boldsymbol{\beta}_{\boldsymbol{LV}_{\mathbf{4}}}$ | Th + CN + Put. + GP | Th + CN + Put. + Hipp. | Th + CN + Put. + Amyg. | Th + CN + Put. + Acc. | Th + CN + GP + Hipp. | Th + CN + GP + Amyg. | Th + CN + GP + Acc. | Th + CN + Hipp. + Amyg. | Th + CN + Hipp. + Acc. | Th + CN + Amyg. + Acc. | Th + Put. + GP + Hipp. | Th + Put. + GP + Amyg. | Th + Put. + GP + Acc. | Th + Put. + Hipp. + Amyg. | Th + Put. + Hipp. + Acc. | Th + Put + Amyg. + Acc. | Th + GP + Hipp. + Amyg. | Th + GP + Hipp. + Acc. | Th + GP + Amyg. + Acc. | Th + Hipp. + Amyg. + Acc. | CN + Put. + GP + Hipp. |
| $\boldsymbol{HLM \sim}\boldsymbol{\beta}_{\boldsymbol{0}}\boldsymbol{+}\boldsymbol{\beta}_{\boldsymbol{LV}_{\mathbf{1}}}\boldsymbol{+}\boldsymbol{\beta}_{\boldsymbol{LV}_{\mathbf{2}}}\boldsymbol{+}\boldsymbol{\beta}_{\boldsymbol{LV}_{\mathbf{3}}}\boldsymbol{+}\boldsymbol{\beta}_{\boldsymbol{LV}_{\mathbf{4}}}\boldsymbol{+}\boldsymbol{\beta}_{\boldsymbol{LV}_{\mathbf{5}}}$ | Th + CN + Put. + GP + Hipp. | Th + CN + Put. + GP + Amyg. | Th + CN + Put. + GP + Acc. | Th + CN + Put. + Hipp. + Amyg. | Th + CN + Put. + Hipp. + Acc. | Th + CN + Put. + Amyg. + Acc. | Th + CN + GP + Hipp. + Amyg. | Th + CN + GP + Hipp. + Acc. | Th + CN + GP + Amyg. + Acc. | Th + CN + Hipp. + Amyg. + Acc. | Th + Put. + GP + Hipp. + Amyg. | Th + Put. + GP + Hipp. + Acc. | Th + Put. + GP + Amyg. + Acc. | Th + Put. + Hipp. + Amyg. + Acc. | Th + GP + Hipp. + Amyg. + Acc. | CN + Put. + GP + Hipp. + Amyg. | CN + Put. + GP + Hipp. + Acc. | CN + Put. + GP + Amyg. + Acc. | CN + Put. + Hipp. + Amyg. + Acc. | CN + GP + Hipp. + Amyg. + Acc. | Put. + GP + Hipp. + Amyg. + Acc. |
| $\boldsymbol{HLM \sim}\boldsymbol{\beta}_{\boldsymbol{0}}\boldsymbol{+}\boldsymbol{\beta}_{\boldsymbol{LV}_{\mathbf{1}}}\boldsymbol{+}\boldsymbol{\beta}_{\boldsymbol{LV}_{\mathbf{2}}}\boldsymbol{+}\boldsymbol{\beta}_{\boldsymbol{LV}_{\mathbf{3}}}\boldsymbol{+}\boldsymbol{\beta}_{\boldsymbol{LV}_{\mathbf{4}}}\boldsymbol{+}\boldsymbol{\beta}_{\boldsymbol{LV}_{\mathbf{5}}}\boldsymbol{+}\boldsymbol{\beta}_{\boldsymbol{LV}_{\mathbf{6}}}$ | Th + CN + Put. + GP + Hipp. + Amyg. | Th + CN + Put. + GP + Hipp. + Acc. | Th + CN + Put. + GP + Amyg. + Acc. | Th + CN + Put. + Hipp. + Amyg. + Acc. | Th + CN + GP + Hipp. + Amyg. + Acc. | Th + Put. + GP + Hipp. + Amyg. + Acc. | CN + Put. + GP + Hipp. + Amyg. + Acc. |  |  |  |  |  |  |  |  |  |  |  |  |  |  |
| $\boldsymbol{HLM \sim}\boldsymbol{\beta}_{\boldsymbol{0}}\boldsymbol{+}\boldsymbol{\beta}_{\boldsymbol{LV}_{\mathbf{1}}}\boldsymbol{+}\boldsymbol{\beta}_{\boldsymbol{LV}_{\mathbf{2}}}\boldsymbol{+}\boldsymbol{\beta}_{\boldsymbol{LV}_{\mathbf{3}}}\boldsymbol{+}\boldsymbol{\beta}_{\boldsymbol{LV}_{\mathbf{4}}}\boldsymbol{+}\boldsymbol{\beta}_{\boldsymbol{LV}_{\mathbf{5}}}\boldsymbol{+}\boldsymbol{\beta}_{\boldsymbol{LV}_{\mathbf{6}}}\boldsymbol{+}\boldsymbol{\beta}_{\boldsymbol{LV}_{\mathbf{7}}}$ | Th + CN + Put. + GP + Hipp. + Amyg. + Acc. |  |  |  |  |  |  |  |  |  |  |  |  |  |  |  |  |  |  |  |  |

| *Regression* | Comb. 22 | Comb. 23 | Comb. 24 | Comb. 25 | Comb. 26 | Comb. 27 | | Comb. 28 | | Comb. 29 | | Comb. 30 | | Comb. 31 | | Comb. 32 | | Comb. 33 | | Comb. 34 | | Comb. 35 | |
| --- | --- | --- | --- | --- | --- | --- | --- | --- | --- | --- | --- | --- | --- | --- | --- | --- | --- | --- | --- | --- | --- | --- | --- |
| $\boldsymbol{HLM \sim}\boldsymbol{\beta}_{\boldsymbol{0}}\boldsymbol{+}\boldsymbol{\beta}_{\boldsymbol{LV}_{\mathbf{1}}}$ | NaN | NaN | NaN | NaN | NaN | | NaN | | NaN | | NaN | | NaN | | NaN | | NaN | | NaN | | NaN | | NaN |
| $\boldsymbol{HLM \sim}\boldsymbol{\beta}_{\boldsymbol{0}}\boldsymbol{+}\boldsymbol{\beta}_{\boldsymbol{LV}_{\mathbf{1}}} \boldsymbol{+}\boldsymbol{\beta}_{\boldsymbol{LV}_{\mathbf{2}}}$ | NaN | NaN | NaN | NaN | NaN | | NaN | | NaN | | NaN | | NaN | | NaN | | NaN | | NaN | | NaN | | NaN |
| $\boldsymbol{HLM \sim}\boldsymbol{\beta}_{\boldsymbol{0}}\boldsymbol{+}\boldsymbol{\beta}_{\boldsymbol{LV}_{\mathbf{1}}}\boldsymbol{+}\boldsymbol{\beta}_{\boldsymbol{LV}_{\mathbf{2}}}\boldsymbol{+}\boldsymbol{\beta}_{\boldsymbol{LV}_{\mathbf{3}}}$ | CN + GP + Acc. | CN + Hipp. + Amyg. | CN + Hipp. + Acc. | CN + Amyg. + Acc. | Put. + GP + Hipp. | | Put. + GP + Amyg. | | Put. + GP + Acc. | | Put. + Hipp. + Amyg. | | Put. + Hipp. + Acc. | | Put. + Amyg. + Acc. | | GP + Hipp. + Amyg. | | GP + Hipp. + Acc. | | GP + Amyg. + Acc. | | Hipp. + Amyg. + Acc. |
| $\boldsymbol{HLM \sim}\boldsymbol{\beta}_{\boldsymbol{0}}\boldsymbol{+}\boldsymbol{\beta}_{\boldsymbol{LV}_{\mathbf{1}}}\boldsymbol{+}\boldsymbol{\beta}_{\boldsymbol{LV}_{\mathbf{2}}}\boldsymbol{+}\boldsymbol{\beta}_{\boldsymbol{LV}_{\mathbf{3}}}\boldsymbol{+}\boldsymbol{\beta}_{\boldsymbol{LV}_{\mathbf{4}}}$ | CN + Put. + GP + Hipp. | CN + Put. + GP + Amyg. | CN + Put. + GP + Acc. | CN + Put. + Hipp. + Amyg. | CN + Put. + Amyg. + Acc. | | CN + GP + Hipp. + Amyg. | | CN + GP + Hipp. + Acc. | | CN + GP + Amyg. + Acc. | | CN + Hipp. + Amyg. + Acc. | | Put. + GP + Hipp. + Amyg | | Put. + GP + Hipp. + Acc. | | Put. + GP + Amyg. + Acc. | | Put. + Hipp. + Amyg. + Acc | | GP + Hipp. + Amyg. + Acc. |
