# Supplementary material for: Modulation of alpha oscillations by attention is predicted by hemispheric asymmetry of subcortical regions
Source: eLife. 2024 Jul 17;12:RP91650. doi: 10.7554/eLife.91650 (PMC11254381; doi:10.7554/eLife.91650)
Supplement: Supplementary file 3. — The Pearson correlation between each subcortical structure with HLM(RIFT) and behavioural asymmetry was calculated. The likelihood of the data under the alternative hypothesis (the evidence of correlation) were subsequently compared to the likelihood under null hypothesis (absence of correlation), given the data. As it is demonstrated in the table, all Bayes factors were below or very close to 1 indicating evidence for the null hypothesis. [file elife-91650-supp3.docx]

Supplementary file 3. Bayes factors for correlation between hemispheric laterality of subcortical structures with hemispheric lateralization modulation of rapid invisible frequency tagging (HLM(RIFT)) and with behavioural asymmetry (BA). The Pearson correlation between each subcortical structure with HLM(RIFT) and behavioural asymmetry was calculated. The likelihood of the data under the alternative hypothesis (the evidence of correlation) were subsequently compared to the likelihood under null hypothesis (absence of correlation), given the data. As it is demonstrated in the table, all Bayes factors were below or very close to 1 indicating evidence for the null hypothesis.

| **Subcortical structure** | **HLM(RIFT)** | **BA** |
| --- | --- | --- |
| Thalamus | 0.14 | 0.20 |
| Caudate Nucleus | 0.18 | 0.15 |
| Putamen | 0.14 | 0.14 |
| Globus Pallidus | 0.34 | 0.13 |
| Hippocampus | 0.19 | 0.25 |
| Amygdala | 0.54 | 1.18 |
| Nucleus Accumbens | 0.16 | 0.17 |
